# Good Agreements Make Good Friends

**DOI:** 10.1038/srep02695

**Published:** 2013-09-18

**Authors:** The Anh Han, Luís Moniz Pereira, Francisco C. Santos, Tom Lenaerts

**Affiliations:** 1 AI lab, Computer Science Department, Vrije Universiteit Brussel, Pleinlaan 2, 1050 Brussels, Belgium; 2 MLG, Département d'Informatique, Université Libre de Bruxelles, Boulevard du Triomphe CP212, 1050 Brussels, Belgium; 3 Centro de Inteligência Artificial (CENTRIA), Departamento de Informática, Faculdade de Ciências e Tecnologia, Universidade Nova de Lisboa, 2829-516 Caparica, Portugal; 4 INESC-ID and Instituto Superior Ténico, Universidade de Lisboa, IST-Taguspark, 2744-016 Porto Salvo, Portugal; 5 ATP-group, CMAF, Instituto para a Investigação Interdisciplinar, P-1649-003 Lisboa Codex, Portugal

## Abstract

When starting a new collaborative endeavor, it pays to establish upfront how strongly your partner commits to the common goal and what compensation can be expected in case the collaboration is violated. Diverse examples in biological and social contexts have demonstrated the pervasiveness of making prior agreements on posterior compensations, suggesting that this behavior could have been shaped by natural selection. Here, we analyze the evolutionary relevance of such a commitment strategy and relate it to the costly punishment strategy, where no prior agreements are made. We show that when the cost of arranging a commitment deal lies within certain limits, substantial levels of cooperation can be achieved. Moreover, these levels are higher than that achieved by simple costly punishment, especially when one insists on sharing the arrangement cost. Not only do we show that good agreements make good friends, agreements based on shared costs result in even better outcomes.

Conventional wisdom suggests that cooperative interactions have a bigger chance of surviving when all participants are aware of the expectations and the possible consequences of their actions. All parties then clearly know to what they commit and can refuse such a commitment whenever the offer is made. A classical example of such an agreement is marriage^1^,^2^. In that case mutual commitment ensures some stability in the relationship, reducing the fear of exploitation and providing security against potential cataclysms. Clearly such agreements can be beneficial in many situations, which are not limited to the type of formal and explicit contracts as is the case for marriage. Commitments may even be arranged in a much more implicit manner as is the case for members of the same religion^3^,^4^, or by some elaborate signaling mechanism as is the case in primates' use of signaling to synchronize expectations and the consequences of defaulting on commitment in their different ventures^5^.

Here we investigate analytically and numerically whether costly commitment strategies, in which players propose, initiate and honor a deal, are viable strategies for the evolution of cooperative behavior, using the symmetric, pairwise, and non-repeated Prisoner's Dilemma (PD) game to model a social dilemma. Next to the traditional cooperate (C) and defect (D) options, a player can propose its co-player to commit to cooperation before playing the PD game, willing to pay a personal cost 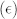 to make it credible. If the co-player accepts the arrangement and also plays C, they both receive their rewards for mutual cooperation. Yet if the co-player plays D, then he or she will have to provide the proposer with a compensation at a personal cost (*δ*). Finally, when the co-player does not accept the deal, the game is not played and hence both obtain no payoff.

Although there is a kind of punishment associated with the agreement, the notion of compensating a partner when not honoring a negotiated deal is not entirely equivalent to the general notion of punishment as has been studied in Evolutionary Game Theory^6^,^7^,^8^,^9^,^10^,^11^,^12^,^13^ so far. In the current work, both parties are aware of the stakes before they start the interaction: the person who accepts to commit knows upfront what to expect from the person that proposes the commitment and what will happen if he or she does not act appropriately. Even more, the co-player has the possibility not to accept such an agreement and continue interacting without any prior commitment with the other players, and with no posterior repercussions from commitment proposers. In the current literature, punishment (with or without cost) is imposed as a result of “bad” behavior, which can only be escaped by not participating in the game at all^9^,^12^,^14^. As such, the present work differs from peer and pool punishment^9^,^12^ in that the latter *imposes* the commitment to the other players, i.e. defectors will always be punished even when they did not want to play with punishers. In addition, there is no notion of compensation incorporated in the model that remunerates the proposer when her accepted deal is violated, in contradistinction to our own model. Moreover, because the creation of the agreement occurs explicitly in the current work, players can behave conditionally (even without considering previous interactions, whether direct or indirect), and that is not considered in the ongoing evolutionary models of peer and pool punishment. However, it is quite plausible to cooperate only when someone asks to commit and to otherwise defect in order to avoid exploitation, even in a one-shot interaction.

Nevertheless, in both punishment and of commitment strategies, a cost 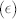 may be associated with the means to punish the other player, as is the case for the former strategy, or for setting-up the agreement in case of the latter strategy. A large body of evidence shows that humans are willing to incur a personal cost in order to punish those who free-ride on the cooperative behavior of themselves or others. Several theoretical and experimental studies demonstrate that such costly punishment (CP) may sustain cooperation even in games without repeated interactions^6^,^7^,^8^,^9^,^11^,^12^,^14^,^15^,^16^,^17^,^18^,^19^,^20^. However, there is also evidence that CP may not be beneficial to cooperation or fails to prevail in populations playing one-shot PD games or public goods games, even when (e.g.) direct reciprocity^21^,^22^,^23^, indirect reciprocity^24^, networked populations^25^ or optional participation^26^ are considered. As such, this work provides an important novel approach for understanding the emergence of cooperative behavior in social dilemmas, in which both commitment and punishment strategies are explicitly (and independently) considered.

One important conclusion derived from recent experiments is that CP may maintain cooperation only if the behavior is cost effective, meaning a low cost 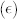 for the punisher and sufficiently significant impact (*δ*) on the punished^16^,^17^,^27^,^28^. As we show later on analytically for the PD game, this ratio may become quite excessive if one wants to obtain (nearly) full cooperation in CP (see also Supplementary Information). Hence one can wonder: Would a prior agreement upon both the compensation and the impact that this compensation has on the defaulting player, lead to a better balance between arrangement cost and compensation? It might be that a high compensation will scare off non-cooperative players and give incentives for the cooperative ones to join forces. However, because arranging it is in itself costly, the ones that free-ride on others' efforts to bring the contract in may fare better than the proposers themselves.

In summary, the main questions we ask here are whether proposing such commitments and honoring them is a viable strategy for the evolution of cooperative behavior and how such a commitment deal should be arranged in order to reach high levels of cooperation. That is, given the game at hand and the potential cost of arranging commitment, is it worth arranging it at all? And if yes, what kind of compensation should be associated with the commitment proposal? For commitment proposers to prevail, they need to justify the cost of arranging the commitment in order to win against the free-riders and the traditional defectors, as well as exhibiting a strong enough penalty for the fake committers. Additionally, we examine how this model of prior mutual commitment differs from CP.

## Results

### Commitment strategies and free-riders

We consider here, next to the traditional pure cooperator (C) and defector (D) strategies, a commitment proposing (COMP) strategy, which proposes a commitment to others, is willing to pay the cost 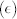 of setting up the deal (if required) and plays C when the opponent accepts the deal. When the deal is not accepted this type of individual refuses to play the game. Yet, since such a commitment proposal provides information about the high incentive to cooperate of the player, other players may exploit that information to gain an advantage over him or her. We encompass these possibilities, considering two additional strategies, which can be considered as two types of second-order free-riders:

- The fake committers (FAKE), who accept a commitment proposal yet defect when playing the game, assuming that they can exploit the COMP players without suffering a severe consequence.
- The commitment free-riders (FREE), who defect unless being proposed a commitment, which they then accept and cooperate afterwards in the PD game. In other words, these players are willing to cooperate when a commitment is proposed but are not prepared to pay the cost of setting it up.

Every individual in the population will adhere to one of these five strategies whilst playing the PD game, where the game is traditionally defined by the following parametrized payoff matrix: 
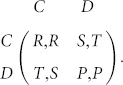


Once the interaction is established and both players have decided to play C or D (with or without commitment arrangements), both players receive the same reward *R* (penalty *P*) for mutual cooperation (mutual defection). Unilateral cooperation provides the sucker's payoff *S* for the cooperative player and the temptation to defect *T* for the defecting one. The payoff matrix corresponds to the preferences associated with the Prisoner's Dilemma when the parameters satisfy the ordering, *T* > *R* > *P* > *S*^29^. A large body of literature has shown that for cooperation to evolve in this kind of social dilemma certain mechanisms like repetition of interactions, reputation effects, kin and group relations or structured populations, need to be introduced^30^,^31^. In the current study we focus only on the strategic behavior of proposing commitments, ignoring for now all those other mechanisms.

Since pure cooperators (C) are always better off to engage in a commitment than otherwise— as cooperation is their default choice and a positive compensation is guaranteed when being exploited—they will always accept to commit when being asked to. On the other hand, pure defectors (D) will never accept commitment proposals since they would otherwise have to compensate the proposer, and as a consequence would be equivalent to the FAKE players. When two COMP players interact only one of them will need to pay the cost of setting up the commitment. Yet, as either one of them can take this action they pay this cost only half of the time. Hence, the payoff awarded to each COMP player is, on average, 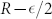. One can interpret this as an implicit form of cost sharing.

Together these five strategies define the following payoff matrix, capturing the average payoffs that each strategy will receive upon interaction with one of the other four strategies 
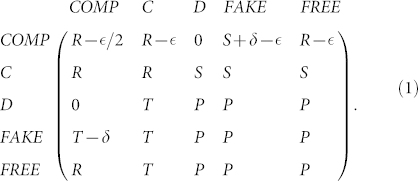
A first quick examination of this matrix already shows that, in a pairwise manner, D, FAKE and FREE players are neutral among themselves, since they all receive P for their pairwise interaction. In addition, C will be advantageous to COMP players since they do not have to pay the commitment cost.

Note that given the current setting there are other possible strategies, such as those that propose commitment but do not cooperate or do not accept commitment proposed by others. These strategies are currently omitted for the sake of exposition, also for they get eliminated anyway since they are dominated by at least one of the strategies in the current model (see Supplementary Information).

### Constraints on the viability of commitment proposers

Whether COMP is a viable strategy depends on the commitment parameters, i.e. the cost of arranging the deal 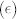 and the compensation cost (*δ*). One can determine this viability analytically by deducing under which conditions COMP is risk-dominant with respect to D, FAKE and FREE players (see Methods). Using the equation (5), the following conditions relative to the commitment parameters 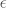 and *δ* can be obtained concerning the risk-dominance of COMP against any of the three strategies D, FAKE and FREE: 
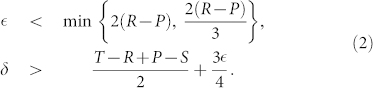
To render these conditions understandable, we reduce here the parameterized PD game to the Donation game^31^, where *T* = *b*, *R* = *b* − *c*, *P* = 0, *S* = −*c* and *b* > *c*, with *b* and *c* representing the benefit and cost of cooperation respectively. As a consequence, the previous conditions are simplified to: 
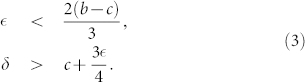
Thus, whenever both 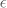 and *δ* meet the conditions listed in equation (3) then COMP is risk-dominant against D, FAKE and FREE. Both conditions can be understood intuitively: to make a successful commitment a COMP player wants to ensure that there is a sufficient benefit in setting up the deal (roughly 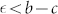) and that when the co-player defaults, she is sufficiently compensated (roughly 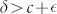) for honoring her side of the deal. When one of these conditions is not met, the COMP player can be exploited (by D and FREE players in case of the first condition and by FAKE players in case of the second) leading the overall population behavior towards increasing levels of defection.

### An agreement is as good as its investment cost

**Figure 1:**
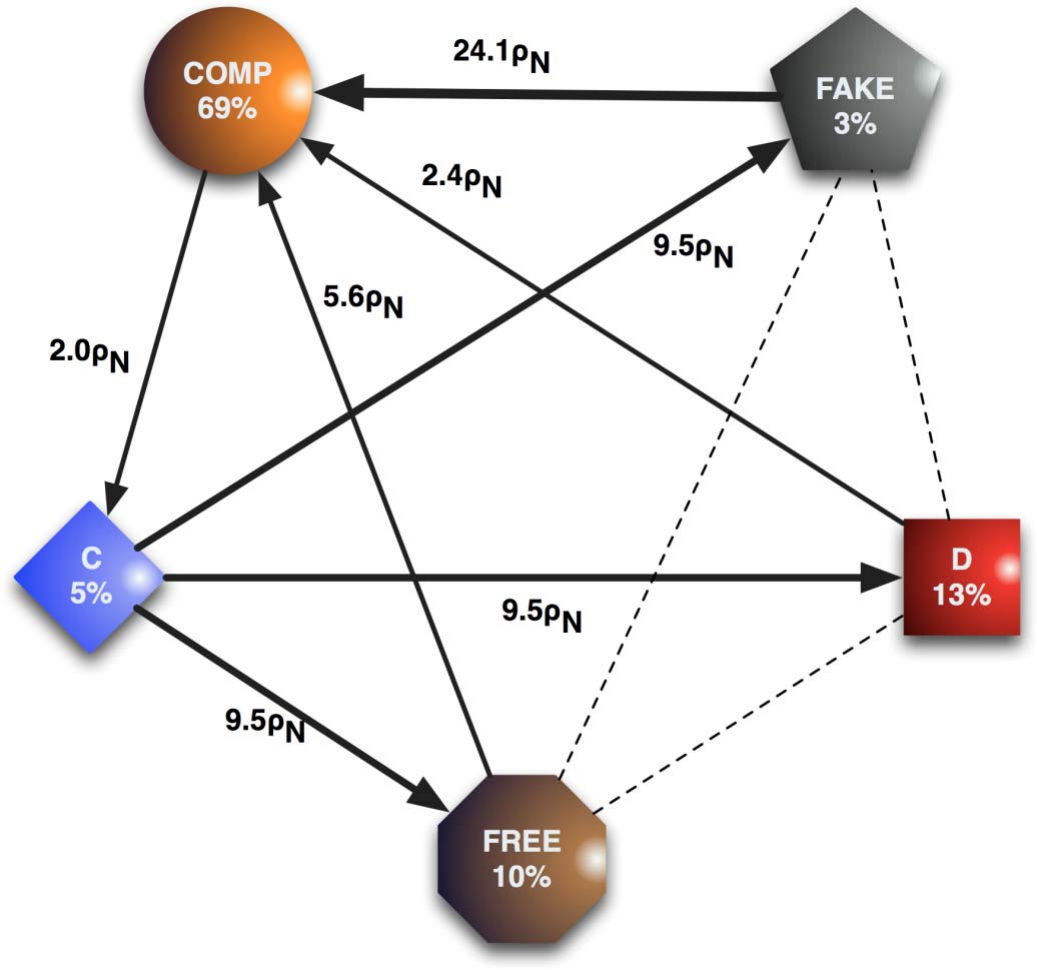
Stationary distribution and fixation probabilities. The population spends most of the time in the homogenous state of COMP. The black arrows identify the advantageous transitions, and the dashed lines stand for neutral transitions. Note the cyclic pattern from cooperative to defection to commitment strategies and back. Parameters: *T* = 2, *R* = 1, *P* = 0, *S* = −1; *δ* = 4; 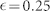; imitation strength, *β* = 0.1; population size, *N* = 100; *ρ_N_* = 1/*N* denotes the neutral fixation probability.

**Figure 2:**
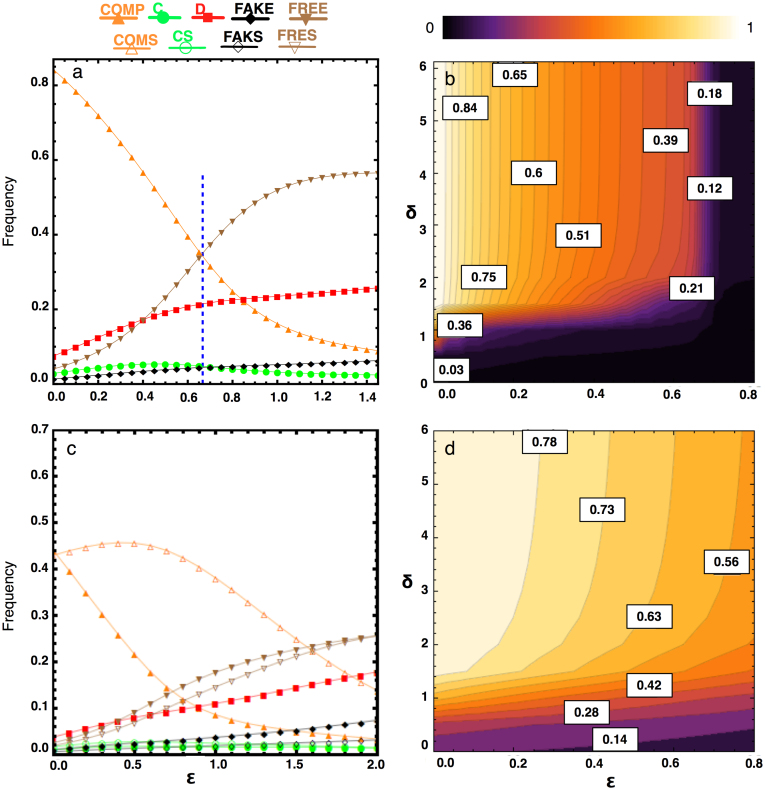
(a) Frequency of the five strategies as a function of 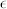. The population spends most of the time in the homogenous state of COMP for small enough 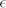. The commitment free-riders (FREE) dominate once the value of 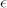 exceeds the boundary 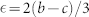 (see dashed vertical blue line) followed by the D players. (b) Frequency of COMP as a function of 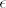 and *δ*. In a population of COMP, C, D, FAKE and FREE individuals, for a wide range of 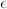 and *δ*, the population spends most of the time in the homogeneous state of COMP. In general, the smaller the cost of proposing commitment, 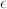, and the greater the compensation for honoring a violated commitment, *δ*, the greater the frequency of COMP. However, for any given 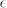, there is a threshold of *δ* where only a very small improvement to the fraction of COMP can be observed by increasing it. (c) This can be improved if the commitment proposers ask co-players to share the cost of arranging commitments (COMS), though all the non-proposing commitment strategies can opt out of playing when being asked to share that cost (see also panel d). COMP is slightly better than COMS if it is approximately cost free to arrange commitments (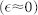) (see also Figures S3c and S3d), because other strategies shy from the additional cost of sharing. (d) Total frequency of commitment strategies, COMS plus COMP, as a function of 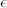 and *δ*. Parameters: *T* = 2, *R* = 1, *P* = 0, *S* = −1; *β* = 0.1; *N* = 100; In panels a and c, *δ* = 4.

These observations become even clearer when considering the fixation probabilities and stationary distributions (see Methods) of the different strategies, as visualized in Figure 1. When the cost of arranging commitment 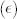 is sufficiently small, the population spends most of the time in the homogeneous state with COMP players (see also Figures 2a and 2b), regardless of the initial composition of the population. Yet, as can also be gleaned from Figure 1, cycles from C to defection strategies (FREE, D and FAKE) and back over to COMP strategists emerge. Nevertheless, under the conditions mentioned earlier, COMP remains the dominant strategy thereby ensuring significant levels of cooperation.

As 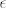 approaches its boundary mentioned in equation (3), see Figure 2a, FREE players, and in their wake the D players, start to rise in number. Once 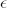 moves beyond the boundary, these defecting types start to dominate the population. FREE players benefit more strongly than D players from the increase in commitment costs: they keep on gaining the reward from the cooperative interaction with the COMP players and do not suffer from the costly overhead for arranging the deal. A population containing only FAKE or C players emerges very rarely, as C strategists are always exploited by the three defecting strategies, and FAKE players suffer from the compensation they need to pay to the COMP players. Additionally, the importance of the game, which scales with the net benefit from cooperation (*b* − *c*), will determine which arrangement cost, 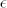, is justified for the game (see Supporting Information, Figure S3).

As shown in Figure 2b, for low 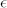, nearly homogeneous COMP populations are almost always reached for sufficiently large *δ*. Yet, more interestingly, this high frequency is not affected by changes in the compensation *δ*, once a certain threshold is reached. Accordingly, the arrangement cost is the essential parameter for the emergence and survival of mutual cooperation in the current extension of the PD game. This effect is absent in case of costly punishment (CP), as can be seen in the Supplementary Information. In that case, the effective punishment *δ* increases with the cost of punishment (see Figure S2). Moreover, to reach the same level of cooperation as in the commitment model a much more severe punishment (*δ* ≈ 14) is required for an equivalently small cost (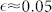). As such, commitment effectively reduces the cost-to-impact ratio. However, for larger 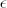 (and also larger *δ*), CP is more effective than COMP because, in the latter, commitment free-riders (FREE) become dominant (see Figure 1), whereas these players are efficiently punished by CP. Still, as will be shown in the next section, this problem may be resolved to a significant extent by sharing the cost of setting-up the deal.

### Explicitly sharing the cost leads to more favourable outcomes

As mentioned earlier, when two COMP players interact they each receive on average 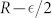, as they both have a 50% chance of paying the cost for setting up the deal. Implicitly, this outcome represents a form of cost sharing between these players. Yet, other players (C, FREE and FAKE) are not as forthcoming, requiring the COMP strategist to cough up the complete amount 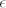. Assume now that a COMP individual can, when proposing a deal, also request her co-player to share the arrangement cost, which we will refer to as a commitment sharing or COMS strategist. Accordingly, the cost sharing becomes explicit now. When encountering such an individual the co-player has two choices to make before playing the game: she has to decide to accept or reject the commitment proposal and when she accepts she has to decide whether she is willing to share the cost or not.

Assuming that all previous commitment-accepting strategies, i.e. C, FAKE and FREE, do not agree to share the cost, three additional strategies that are prepared to share the cost once this is proposed, need to be introduced. We will refer to these strategies as CS, FAKS and FRES, respectively (see Supplementary information for the payoff matrix).

As was done for COMP individuals previously (see Constraints on the viability of commitment proposers), the conditions for which COMS strategists are risk-dominant against all defectors and free-riders (i.e. D, FAKE, FAKS, FREE and FRES) can be determined: 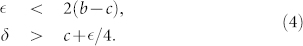
Comparing to equation (3), one can see that the conditions for the viability of COMS are less restrictive than those derived for COMP: On the one hand, a bigger arrangement cost 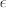 (up to 2(*b* − *c*) instead of 2(*b* − *c*)/3) is allowed for commitment proposers to still be risk-dominant. On the other hand, a smaller compensation is required to be successful against individuals playing the FAKE and FAKS strategies (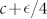 compared to 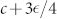). Both Figure 2C and Figure 2d clarify this observation further, showing that COMS is a viable evolutionary strategy for a wider range of 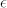. Even for a larger cost, the total frequency of commitment proposers (both COMS and COMP) benefit now from a higher *δ*, while asking their co-players to share the cost of arranging commitments. This way the commitment free-riders can either share the cost (FRES and FAKS), or opt for not playing (FREE and FAKE), hence resolving the commitment free-riding issue.

In the literature of costly punishment, pool punishment is an effective solution towards the problem of second-order free riding, where cooperation can be subverted if those who contribute to the joint effort but do not punish defectors, are not punished themselves^12^,^14^. As the cost is shared among pool punishers, they may generate an impact on the punished more significant than what they can do by themselves. However, a population of pool punishers has a lower average payoff than that of peer punishers, because pool punishers pay in advance while the latter do so reactively, only when facing a defecting act. That is, ‘efficiency is traded for stability'^12^.

Differently, in our commitment model, sharing costs does not reduce the efficiency. It is tantamount to ‘*generosity is traded for stability*', assuming COMP is deemed more generous than COMS as it voluntarily pays the whole arrangement cost, expecting to attract more (non-fake) committers. We envisage that COMP may do better if reputation effects are considered, assigning higher reputation for COMP than COMS. Otherwise, it is better off proposing cost-sharing deals.

## Discussion

We show in this paper, in the context of the one-shot pairwise Prisoner's Dilemma, that even in the absence of repeated interactions, reputation effects, network reciprocity, as well as group and kin selection, asking to commit and spelling out of the consequences of not honoring the deal is an effective mechanism promoting the emergence of cooperation. If commitment can be arranged in such a way that its cost is justified with respect to the benefit of cooperation (roughly up to the payoff of mutual cooperation), by associating a sufficient compensation (roughly equal to this cost plus the cost of cooperation), commitment proposers pervade and dominate the population. On the one hand, such commitment proposers can get rid of those individuals that agree to cooperate yet act differently (fake committers) as well as avoid interacting, without any assurance of not being exploited, with the traditional defectors (D). On the other hand, they can maintain a sufficient advantage over those individuals that only cooperate when being asked to commit, because a commitment proposer will cooperate with players alike herself, while the latter defect among themselves. These results suggest our specialized capacity for commitment might have been shaped by natural selection^1^,^32^,^33^,^34^,^35^, together with the evolution of complex signaling systems and associated meanings^36^,^37^.

When the cost of arranging commitments is increased and goes beyond the two conditions mentioned earlier, this advantage vanishes, and the commitment proposers become exploited by free-riders, thereby leading to the disappearance of cooperation. We have shown that this commitment free-riding issue can be dealt with by insisting explicitly on sharing the arrangement cost. Even when the free-riders still can refuse to share, *generosity is traded for stability*. More precisely, when the option of sharing the cost is in place, cooperation is sustained for a much wider range of the parameters. Our model thus demonstrates that in societies with an adequate support for arranging shared costly commitments and resolving with compensation conflicts that follow from not honoring prior commitments, cooperation becomes widespread.

However, what happens when the society is not capable of resolving the conflict? In other words, what if a FAKE player can sometimes get away with cheating? In this case a COMP (or COMS) player would obtain an average compensation smaller than *δ*, for instance proportional to the probability *p* of receiving the compensation, i.e. *p* × *δ*. Considering again the conditions shown in equation 3 (or 4), COMP (or COMS) remains viable as long as the adjusted compensation meets the restraints provided by these equations.

We have also highlighted the differences between the notions of costly commitment and costly punishment, where the latter has been abundantly described in the game theoretical literature^6^,^8^,^9^,^10^,^11^,^12^,^13^. As long as the arrangement cost is within bounds, strategies based on prior commitment will lead to much higher levels of cooperation than those obtained by costly punishment. Moreover, because punishment is a one-sided decision, antisocial punishments, i.e. punishment imposed on ‘good' players, including cooperators and altruistic punishers, may evolve, hence hindering the evolution of cooperation^18^,^26^. Commitments do not lead to this kind of antisocial behaviors: only those who agree to commit can be punished for their wrongdoing. Clearly, even when cooperation based on commitment may lead to considerably better outcomes than that based on costly punishment, in a number of ways, this does not mean that our model replaces the existing ones. They rather complement one another, requiring additional studies examining the balance between both mechanisms in the evolution of cooperative behavior.

Here we have analyzed commitments in the one-shot PD game. Clearly, as can be derived from some of the examples, commitments can be established for longer time periods and may be influenced by reputation effects^1^. In case of the latter, one may decide to break an ongoing commitment when a partner attains a bad reputation. When considering the former, one also needs to take into account that commitments are often mutually exclusive. Hence, future work on studying commitments should be directed to iterated games in combination with other aspects such as direct and indirect reciprocity. Within these extended contexts, an ability to recognize intentions of others based on their prior interactions^38^,^39^,^40^,^41^,^42^ may allow individuals to avoid the cost of arranging commitments (in a transparent world where mutual intentions are easily recognizable, arranging costly commitments is unnecessary), especially when the cost is high^35^,^43^. Another pertinent question is whether cooperation induced by a system of prior commitments may be more stable in case of noise (i.e. mistakes) as for instance for well-known strategies like Tit-for-Tat or Win-Stay-Loose-Shift^31^. In this context, as we have recently shown, apology will play an essential role to render the strategy robust against noise^44^.

In short, our work demonstrates that the conventional wisdom or common knowledge stating that *good agreements make good friends* provides a highly relevant guideline which, as we have shown here, is extendable to *good cost-sharing agreements lead to even better friends*.

## Methods

### Risk-dominant strategies

An important analytical criteria to determine the viability of a given strategy is whether it is risk-dominant with respect to other strategies^30^. Namely, one considers which selection direction is more probable: an A mutant fixating in a homogeneous population of individuals playing B or a B mutant fixating in a homogeneous population of individuals playing A. When the first is more likely than the latter, A is said to be *risk-dominant* against B^30^,^45^,^46^, which holds for any intensity of selection and in the limit of large *N* when 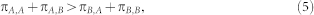
where *π_X,Y_* stands for the payoff an individual using strategy *X* obtained in an interaction with another individual using strategy *Y* (as given in the payoff matrix (1)).

### Evolutionary dynamics in finite populations

Both the analytical and numerical results obtained here use Evolutionary Game Theory methods for finite populations^31^,^47^,^48^. In such a setting, individuals' payoff represents their *fitness* or social *success*, and evolutionary dynamics is shaped by social learning^31^,^49^,^50^, whereby the most successful individuals will tend to be imitated more often by the others. In the current work, social learning is modeled using the so-called pairwise comparison rule^51^, assuming that an individual *A* with fitness *f_A_* adopts the strategy of another individual *B* with fitness *f_B_* with probability *p* given by the Fermi function, 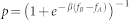. The parameter *β* represents the ‘imitation strength' or ‘intensity of selection', i.e., how strongly the individuals base their decision to imitate on fitness comparison. For *β* = 0, we obtain the limit of neutral drift – the imitation decision is random. For large *β*, imitation becomes increasingly deterministic.

In the absence of mutations or exploration, the end states of evolution are inevitably monomorphic: once such a state is reached, it cannot be escaped through imitation. We thus further assume that, with a certain mutation probability, an individual switches randomly to a different strategy without imitating another individual. In the limit of small mutation rates, the dynamics will proceed with, at most, two strategies in the population, such that the behavioral dynamics can be conveniently described by a Markov Chain, where each state represents a monomorphic population, whereas the transition probabilities are given by the fixation probability of a single mutant^47^,^48^,^52^. The resulting Markov Chain has a stationary distribution, which characterizes the average time the population spends in each of these monomorphic end states.

Let *N* be the size of the population. Suppose there are at most two strategies in the population, say, *k* individuals using strategy A (0 ≤ *k* ≤ *N*) and (*N* − *k*) individuals using strategies B. Thus, the (average) payoff of the individual that uses A and B can be written as follows, respectively, 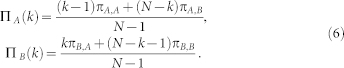
Now, the probability to change the number *k* of individuals using strategy A by ± one in each time step can be written as^51^

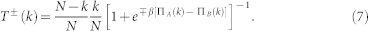
The fixation probability of a single mutant with a strategy A in a population of (*N* − 1) individuals using B is given by^47^,^51^,^53^

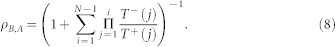
In the limit of neutral selection (i.e. *β* = 0), *ρ_B,A_* equals the inverse of population size, 1/*N*.

Considering a set {1, …, *q*} of different strategies, these fixation probabilities determine a transition matrix 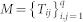, with *T_ij,j≠i_* = *ρ_ji_*/(*q* − 1) and 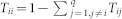, of a Markov Chain. The normalized eigenvector associated with the eigenvalue 1 of the transposed of *M* provides the stationary distribution described above^48^,^52^,^53^, describing the relative time the population spends adopting each of the strategies.

## Author Contributions

T.A.H., L.M.P., F.C.S. and T.L. designed the research. The models were implemented by T.A.H. Results were analyzed and improved by T.A.H., L.M.P., F.C.S. and T.L. T.A.H., L.M.P., F.C.S. and T.L. wrote the paper together.

## Supplementary Material

Supplementary Information Supplementary Information
