# Supplementary material for: Good Agreements Make Good Friends
Source: Sci Rep. 2013 Sep 18;3:2695. doi: 10.1038/srep02695 (PMC3776200; doi:10.1038/srep02695)
Supplement: Supplementary Information [file srep02695-s1.pdf]

Supplementary Information: Good Agreements Make Good Friends

The Anh Han[α,β,*], Luís Moniz Pereira[γ], Francisco C. Santos[δ,ϵ] and Tom Lenaerts[α,β,*]

August 22, 2013

[α] AI lab, Computer Science Department, Vrije Universiteit Brussel, Pleinlaan 2, 1050 Brussels, Belgium

[β] MLG, Département d'Informatique, Université Libre de Bruxelles, Boulevard du Triomphe CP212, 1050 Brussels, Belgium

[γ] Centro de Inteligência Artificial (CENTRIA), Departamento de Informática, Faculdade de Ciências e Tecnologia, Universidade Nova de Lisboa, 2829-516 Caparica, Portugal

[δ] INESC-ID and Instituto Superior Técnico, Universidade de Lisboa, IST-Taguspark, 2744-016 Porto Salvo, Portugal

[ϵ] ATP-group, CMAF, Instituto para a Investigação Interdisciplinar, P-1649-003 Lisboa Codex, Portugal

*corresponding authors: h.anh@ai.vub.ac.be, Tom.Lenaerts@ulb.ac.be

1 Analysis for the full set of strategies

In the main text, we have considered the subset of five most reasonable strategies, COMP, C, D, FAKE and FREE. Some other strategies have been omitted for the sake of exposition. Our reasoning was that those who propose commitments at a cost should cooperate because otherwise (i.e. they defect, thus may also incur the compensation cost) they are worse than the corresponding defectors. Furthermore, those who will cooperate (with or without commitments, i.e. C and COMP) when playing the PD should accept commitments when being asked to because cooperation is their default choice then and a positive compensation is guaranteed plus that they do not have to pay the cost.

Here, in order to achieve a full account of the role of the commitment proposal mechanism for the evolution of cooperation in the one-shot Prisoner's Dilemma, we include all possible strategies (that are not conditional on the existence of a deal as is the case for FREE) given the option to propose commitments.

Let us assume a strategy can be denoted by three components, XYZ, where

- X defines whether the strategy proposes (P) or does not propose (N) a commitment before an interaction ($X \in \{P, N\}$).
- Y defines what is the move, cooperates (C) or defects (D), in the current interaction ($Y \in \{C, D\}$).
- Z defines if the strategy accepts (A) or rejects (R) a commitment deal when being asked to ($Z \in \{A, R\}$).

There are thus eight possible such strategies. The strategy COMP is identical to PCA, that is, proposes a commitment deal before an interaction, cooperates in the current interaction, and accepts when being proposed to commit. Similarly, the strategy C is identical to NCA, FAKE is identical to NDA and D is identical to NDR.

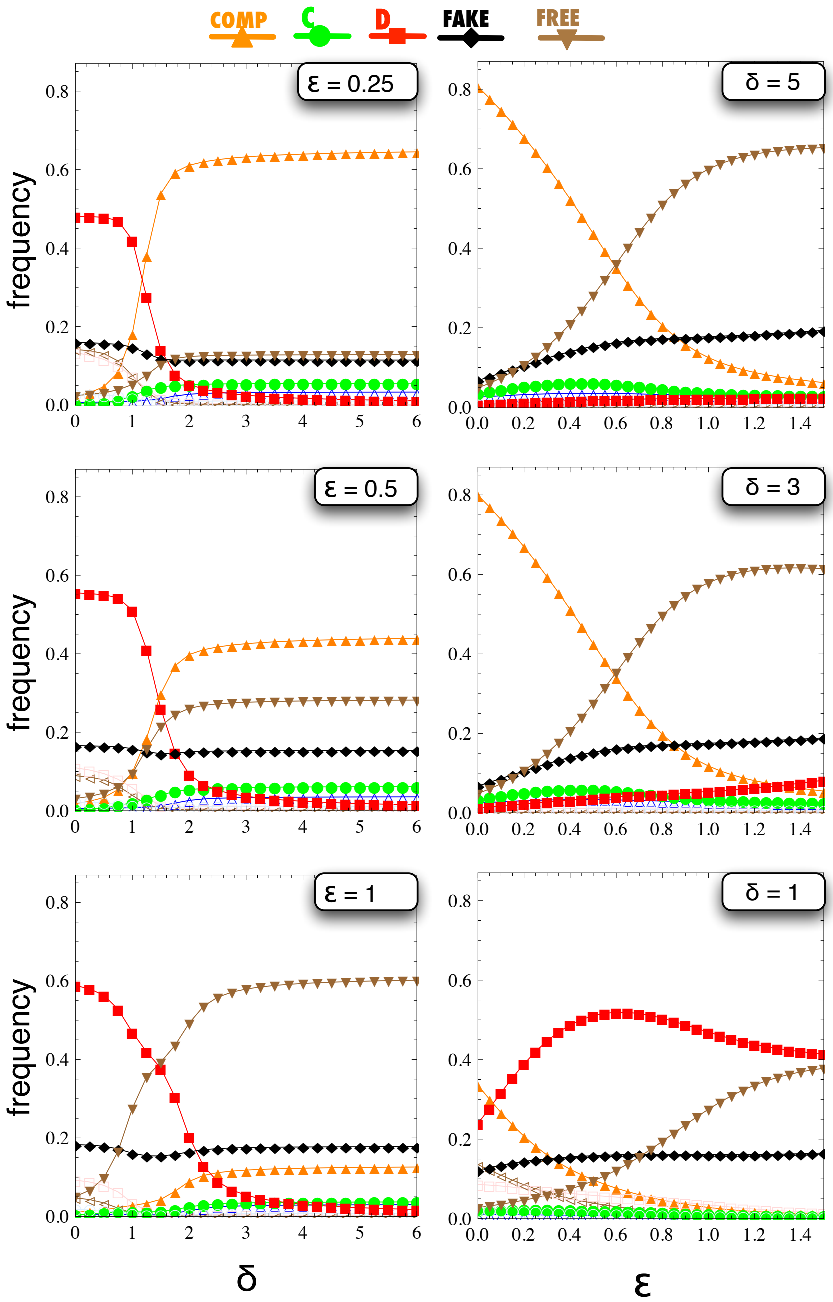

Figure S1: Frequency of each strategy in the population consisting of the 9 strategies. (*Left column*) as a function of δ; and (*Right column*) as a function of ϵ. The five strategies described in the model in the main text, COMP, C, D, FAKE and FREE, always accommodate the significant part of the population, which we highlight for the sake of clarity. The other four strategies have lighter colors. Parameters: $T = 2,\ R = 1,\ P = 0,\ S = -1, N = 100, \beta = 0.1.$

Together with FREE, the average payoff matrix of the nine strategies become

$$
\begin{array}{c} \\ COMP \\ PCR \\ PDA \\ PDR \\ C \\ NCR \\ FAKE \\ D \\ FREE \end{array}
\begin{array}{c}
\begin{array}{ccccccccc} COMP & PCR & PDA & PDR & C & NCR & FAKE & D & FREE \end{array} \\
\begin{pmatrix}
R-\frac{\epsilon}{2} & \frac{R}{2} & S+\delta-\frac{\epsilon}{2} & \frac{S+\delta}{2} & R-\epsilon & 0 & S+\delta-\epsilon & 0 & R-\epsilon \\
\frac{R-\epsilon}{2} & 0 & \frac{1}{2}(S+\delta-\epsilon) & 0 & R-\epsilon & 0 & S+\delta-\epsilon & 0 & R-\epsilon \\
T-\delta-\frac{\epsilon}{2} & \frac{T-\delta}{2} & P-\frac{\epsilon}{2} & \frac{P}{2} & T-\delta-\epsilon & 0 & P-\epsilon & 0 & T-\delta-\epsilon \\
\frac{1}{2}(T-\delta-\epsilon) & 0 & \frac{P-\epsilon}{2} & 0 & T-\delta-\epsilon & 0 & P-\epsilon & 0 & T-\delta-\epsilon \\
R & R & S+\delta & S+\delta & R & R & S & S & S \\
0 & 0 & 0 & 0 & R & R & S & S & S \\
T-\delta & T-\delta & P & P & T & T & P & P & P \\
0 & 0 & 0 & 0 & T & T & P & P & P \\
R & R & S+\delta & S+\delta & T & T & P & P & P
\end{pmatrix}
\end{array}
\quad (1)
$$

From the payoff matrix it becomes clear that PCR is dominated by COMP and NCR is dominated by C (as long as $\delta + S \geq 0$) (a strategy B is dominated by another strategy A if choosing B never gives a better outcome than choosing A, whatever strategy their opponent uses). We hence, as in the main text, assume that those who will/intend to cooperate would never reject a commitment proposal. That is, PCR and NCR can explicitly be removed from the population and the matrix above. In the reduced matrix PDA is dominated by FAKE, and then after removing PDA, PDR is dominated by D (in both dominance cases it holds as long as $\delta \geq T - P - \epsilon$; in the absence of FREE they always holds). Note that the latter dominance holds even in the non-reduced original matrix whenever $\delta \geq T - \epsilon$ and $P \geq 0$. Hence, we can, again as in the main text, assume that those will/intend to defect would not propose commitments.

The following figure clarifies further our results. In Figure S1 we provide numerical results for different combinations of δ and ϵ. It confirms the results obtained in the main text, where the subset of five strategies COMP, C, D, FAKE and FREE are considered. These strategies always consists of the significant part of the whole population. The other four strategies always have very small frequencies.

2 Costly punishment in the Prisoner's Dilemma game

A costly punishment strategy, CP, in the one-shot Prisoner's Dilemma (PD) game, cooperates with her co-player. If the co-player defected she will punish the co-player with a penalty (δ) while suffering a personal cost ϵ. Thus when playing against either a pure cooperator C or pure defector D in the PD game, the following payoff matrix is used:

$$\begin{array}{cc} & \begin{array}{ccc} CP & C & D \end{array} \\ \begin{array}{c} CP \\ C \\ D \end{array} & \begin{pmatrix} R & R & S-\epsilon \\ R & R & S \\ T-\delta & T & P \end{pmatrix} \end{array}. \tag{2}$$

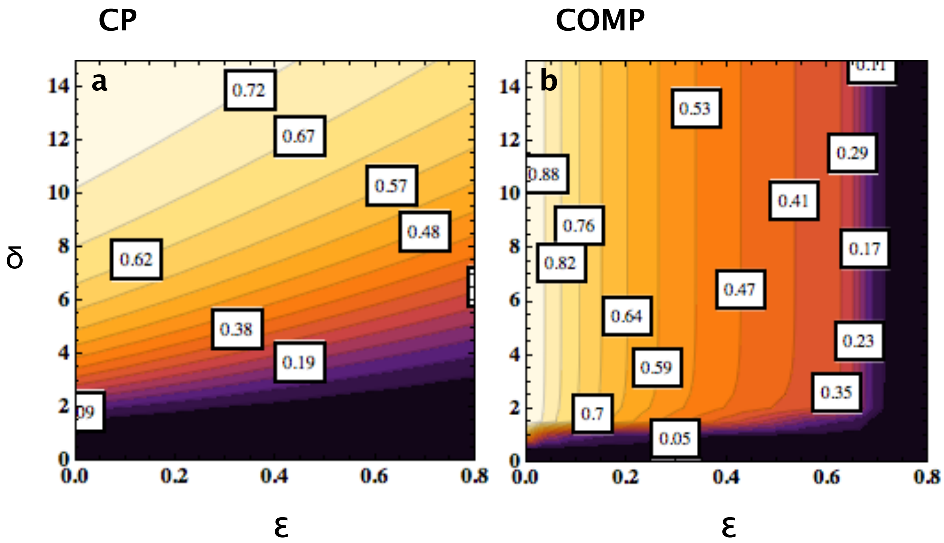

Figure S2: Costly punishment (CP) versus commitment proposal (COMP). (a) fraction of CP in a population with pure C and D strategies; **(b)** fraction of COMP in a population with C, D, FAKE, and FREE players. Parameters: $T = 2,\ R = 1,\ P = 0,\ S = -1$; $\beta = 0.1$; $N = 100$.

Figure S2 shows that, differently from the commitment model where ϵ is the essential/decisive parameter, the effective punishment δ increases with the cost of punishment. Moreover, to reach the same level of cooperation as in the commitment model a much more severe punishment

($\delta \approx 14$) is required for an equivalently small cost ($\epsilon \approx 0.05$).

3 Sharing strategies

Combing all five earlier strategies, the commitment sharing proposer and the three new sharing strategies, the average payoff matrix now becomes

$$
\begin{array}{c} \\ COMS \\ COMP \\ CS \\ C \\ D \\ FAKS \\ FAKE \\ FRES \\ FREE \end{array}
\begin{array}{c}
\begin{array}{ccccccccc} COMS & COMP & CS & C & D & FAKS & FAKE & FRES & FREE \end{array} \\
\left(\begin{array}{ccccccccc}
R-\epsilon/2 & R-\epsilon/2 & R-\epsilon/2 & 0 & 0 & S+\delta-\epsilon/2 & 0 & R-\epsilon/2 & 0 \\
R-\epsilon/2 & R-\epsilon/2 & R-\epsilon & R-\epsilon & 0 & S+\delta-\epsilon & S+\delta-\epsilon & R-\epsilon & R-\epsilon \\
R-\epsilon/2 & R & R & R & S & S & S & S & S \\
0 & R & R & R & S & S & S & S & S \\
0 & 0 & T & T & P & P & P & P & P \\
T-\delta-\epsilon/2 & T-\delta & T & T & P & P & P & P & P \\
0 & T-\delta & T & T & P & P & P & P & P \\
R-\epsilon/2 & R & T & T & P & P & P & P & P \\
0 & R & T & T & P & P & P & P & P
\end{array}\right)
\end{array}. \tag{3}
$$

Using the Equation (5) in the main text, we obtain that COMS is risk-dominant against all defectors and free-riders (i.e. D, FAKE, FAKS, FREE and FRES) if

$$
\begin{aligned}
\epsilon &< 2(R-P) \\
\delta &> \tfrac{T-R+P-S}{2} + \tfrac{\epsilon}{4}.
\end{aligned} \tag{4}
$$

As a consequence, the previous conditions are simplified to:

$$
\begin{aligned}
\epsilon &< 2(b-c), \\
\delta &> c + \tfrac{\epsilon}{4}.
\end{aligned} \tag{5}
$$

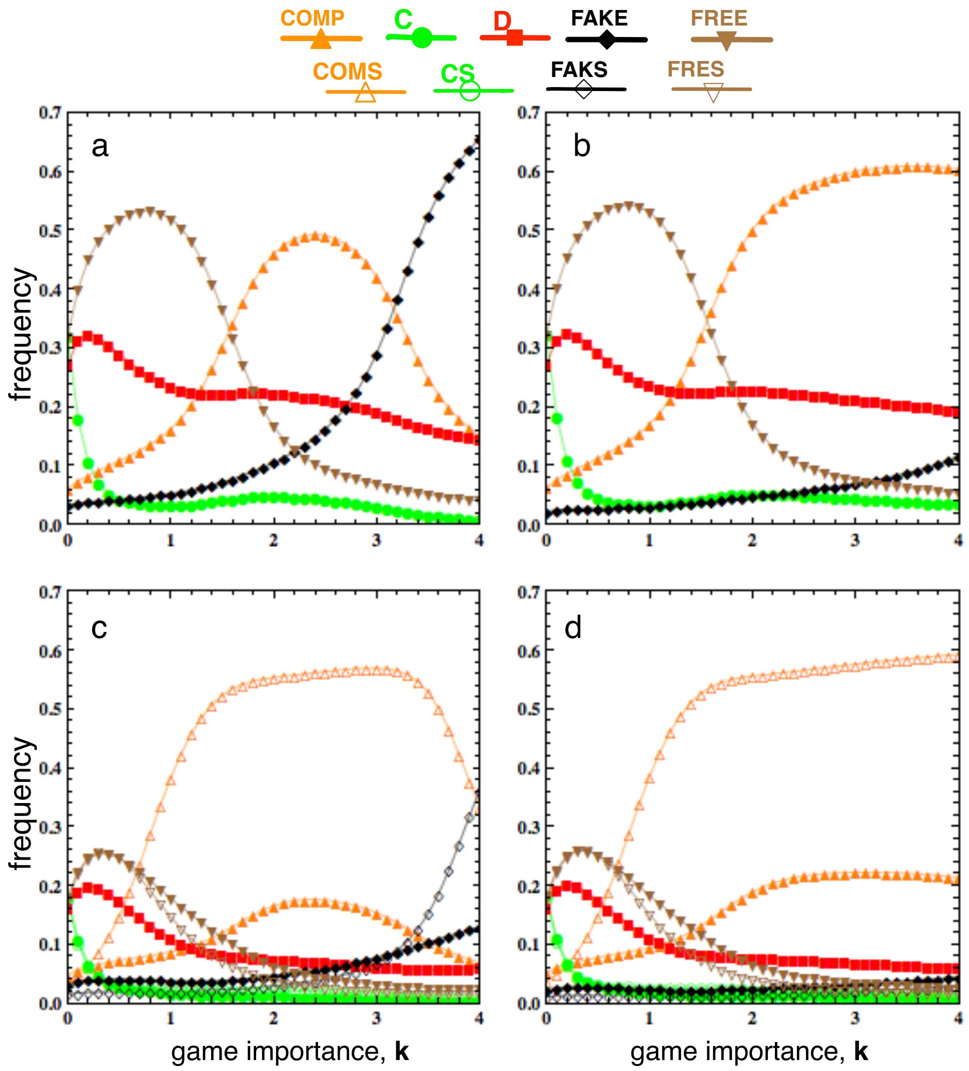

Figure S3: Stationary distribution as a function of k, the importance of the game $(T = 2k, R = k, P = 0, S = -k)$, in a population of five strategies in equation 1 (panels a and b) and in a population of nine strategies in equation (9) (panels c and d), see main text. In the first population, for fixed δ and ϵ, commitment proposers COMP prevail when k is large enough to justify the cost spent arranging the commitment deal, but not too large so that the punishment is not strong enough to deter the fake committers FAKE (in this case, a larger δ should be associated with the commitment deal, as in panel b). Similar behavior is seen for COMS in the second population, although COMS dominates for a much larger range of k. Parameters: (panels a and c) $\delta = 4$ and (panels b and d) $\delta = 6$; in all cases, $\epsilon = 1.0$, $\beta = 0.1$ and $N = 100$.

4 Relevance of commitment arrangement for games of differing importance

Although there is a clear benefit from arranging a commitment, the question remains whether such a deal should be initiated for every interaction. Clearly, when arranging a commitment deal, it is important to justify the cost to arrange it with respect to the importance of the deal at hand, for which the commitment is supposed to help enforce cooperation. In the context of the PD game, one can vary the importance of the game by scaling the payoff each strategy obtains from the interaction, which can be obtained through the introduction of a parameter k. The factor k alters the importance of the PD game in the following manner: $T = 2k,\ R = k,\ P = 0,\ S = -k$ (for the Donation game: $b = 2k$ and $c = k$).

Given this parameterized PD game and assuming a fixed commitment cost ϵ, we ask the question: In which PD games COMP (and later COMS) is a viable strategy? Figures S3a and S3b show, for small values of k, that FREE individuals easily exploit COMP individuals since they are not required to pay the arrangement cost. Once the game becomes important enough relative to the given commitment cost, COMP players prevail. Nevertheless, when the game importance increases further the compensation value is not strong enough to deter the fake committers (FAKE), see Figure S3a, but that can be resolved by requiring a larger compensation δ (Figure S3b). These results clearly comply with the conditions specified earlier in equation (5).

These observations remain the same when including also the sharing strategies COMS, CS, FAKS and FRES, see Figures S3c and S3d. Yet, with the same settings for the parameters ϵ and δ, the commitment proposers still become prevalent for a wider range of PD games, showing again the usefulness of cost-sharing as a viable evolutionary strategy.
